# Combining postgraduate research training, public engagement, and primary school science education—a Superbugs Master (MSc) class

**DOI:** 10.3389/fmicb.2024.1380045

**Published:** 2024-05-31

**Authors:** Jonathan Mark Tyrrell, Haritha Udayan Ayanikkad, Vasudev Nalleppillil-Gopakumar, Rachel Oyebode, Chiamaka Nnamdi Blessing, Sarah Hatch, Matthias Eberl

**Affiliations:** ^1^Institute of Life Science, School of Medicine, Swansea University, Swansea, United Kingdom; ^2^Public Involvement and Engagement Team, School of Medicine, Cardiff University, Cardiff, United Kingdom; ^3^Division of Infection and Immunity, School of Medicine, Cardiff University, Cardiff, United Kingdom; ^4^Systems Immunity Research Institute, Cardiff University, Cardiff, United Kingdom

**Keywords:** public engagement, school education, antimicrobial resistance, microbiology, infection, hygiene

## Abstract

Since 2018, the ‘Superbugs’ initiative at Cardiff University (United Kingdom) has been delivering projects that take a research-driven approach to public engagement, involving rigorous evaluation of the methodologies of delivery and the mechanics of communication. The overall aim of Superbugs is to raise awareness and improve public knowledge of microbiology, infection and antimicrobial resistance (AMR). In the present project, four postgraduate students were recruited to undertake research projects as part of their Master of Science (MSc) studies. After a period of literature appraisals, the students chose to focus on the topic of personal and food hygiene and were tasked with collecting information on effective strategies for educating young children. Taking advantage of a focus group of primary school teachers, the students then designed evidence-informed educational activities and the evaluation strategies by which the impact of these would be assessed. A pilot delivery of these activities was carried out in a community setting at a local public library, before final delivery as part of a school outreach workshop. The MSc students produced three new elements of educational material; a story book, a treasure hunt and an interactive card game, primarily built around the concepts of challenge and gamification. Feedback collected from primary school pupils aged 6–7 years old and teachers indicated that the activities developed were successful in both being engaging to young people and resulting in an improved knowledge on the chosen topics. Taken together, we present evidence that postgraduate research training, underpinned by active and service learning, represents a valid and effective way of delivering impactful public engagement. In turn, the experience holds benefit for the students not only in terms of their academic study and core scientific skills, but also their wider appreciation and confidence in being effective engagers and science communicators.

## 1 Introduction

Public engagement describes the myriad of ways in which the activity and benefits of higher education and research can be shared with the public, according to the UK National Co-ordinating Centre for Public Engagement (Eberl et al., 2023). By definition, it is a two-way process, involving interaction and listening, with the goal of generating mutual benefit. However, the most commonly identified barrier to meaningful and impactful public engagement in scientific research has been the lack of standardisation in mode of delivery and evaluation (Mahony and Stephansen, 2017). Coinciding with that is the difficulty in recruiting members of the academic community to commit to public engagement (Varner, 2014). The use of both undergraduate and postgraduate students has long been a reliable method of populating these activities—albeit often simply as conduits for delivery. We ourselves showed how student volunteers can be called upon to maximum effect in a large-scale scientific pop-up shop in the middle of a busy shopping centre (Tyrrell et al., 2022). However, what has not been fully investigated is a more intrinsic use of students embedded within all aspects of research-drive public engagement: conceptualisation, design, implementation and evaluation.

It could be said that at their core, public engagement and higher education are similar. In both cases, there is an attempt to communicate important and often complex information about a particular topic, in a way that the target audience understands—and in doing so create positive impact allowing the stakeholders (be it the general public or university students) to carry out informed decisions and behaviours. *‘Active learning’* is a teaching philosophy defined as ‘instructional activities involving students in doing things and thinking about what they are doing’ (Bonwell and Eision, 1991). Whilst many barriers to truly effective application of active learning exist (Borte et al., 2020), there is no doubting the positive impacts it can have (Freeman et al., 2014). In correlation to active learning, *‘service learning’* is an often under-utilised term, defined as a student educational experience in which they participate in an organised service activity that meets identified community needs, whilst reflecting to gain further understanding (Bringle and Hatcher, 1995). According to Felten and Clayton (2011), service-based learning is a ‘mechanism for community engagement and high-impact pedagogy’. Applying these teaching approaches in the context of student-driven research projects can result in high quality and impactful public engagement (Webb, 2016; Fatton et al., 2021).

Whilst many academic outreach activities are typically delivered by or with the help of student volunteers, we here explored the potential to formally embed public engagement directly in the training of postgraduate research students, using the Master of Science (MSc) degree in Biomedical Science (BMS) at Swansea University (Wales, United Kingdom) as proof of concept. This was done in the context of the ‘Superbugs’ initiative, a collaboration between academics and public engagement professionals at Swansea University and Cardiff University that aspires to take a research-driven approach to public engagement and facilitate a better understanding of the dynamics and mechanics of positive messaging around microbiology, infection and antimicrobial resistance (AMR; Tyrrell et al., 2022, 2024). Engaging and educating the public on these topics was a cornerstone of the 20-year vision for antimicrobial resistance outlined by the UK Government in 2019 (GOV.UK, 2019), and remains a top priority to date.^1^

We describe the delivery of public engagement and educational activities on the topics of AMR and microbiology through postgraduate student-led research projects; an evaluation of the success of materials produced by this approach in imparting positive impact on targeted stakeholders; an assessment of the efficacy of this approach as a method of delivering such activities; and an evaluation of the impact of the research project on the MSc students themselves.

## 2 Methods

### 2.1 Project aims and participants

Four postgraduate students (HUA, VNG, RO and CNB) contributed to this study as part of their 12-week research projects, forming 60 credits of their Master of Science (MSc) in Biomedical Science (BMS) degree (Clinical Microbiology Specialism) at Swansea University. The projects entailed the students developing both in-person and online educational activities to be delivered in primary school workshops, by utilising and building upon expertise gained and materials developed previously (Tyrrell et al., 2022, 2024). Formal assessment of the students’ work came via a project proposal, delivery of the project, submission of a written dissertation, and an oral presentation. Two of the MSc students were partnered together to design educational activities for in-person delivery, the two other MSc students were partnered to create complementary digital content to be hosted on the *Superbugs.online* educational platform.^2^ The student projects took place between May and August 2023. Two classes of Year 2 pupils (aged 6–7 years old) in Cefn Glas Infants School, an English-medium public primary school in Bridgend (Wales, United Kingdom), were recruited as targeted stakeholders.

### 2.2 Fact-finding and initial design of activities

After a period of independent appraisal of the literature, the MSc students chose personal and food hygiene as their specific area of interest within the wider remit of microbiology, infection and AMR that Superbugs focuses upon. Questionnaires were designed to collect information from teachers and others involved in the supervision, education and engagement of young people. Questions ranged from the need and relevance of educational materials focusing on personal and food hygiene, the methods by which these topics are currently taught, and the interest/success of these on current pupils (Marshall, 2005). The questionnaires were designed in Microsoft Forms and disseminated via Twitter/X, by direct messaging of followers who self-identified as UK-based science teachers in their biographies, taking advantage of the extensive communication network of science and education-related contacts amassed by Superbugs (>1,800 Twitter/X followers by August 2023). The full content of these questionnaires is presented in Supplementary Tables S1, S2.

### 2.3 First delivery of activities

The information collected from the questionnaires and review of existing AMR/microbiology public engagement activities were used to inform the nature and design of the educational activities. After a period of development, an initial draft version of these activities was delivered at a 2 h-long Superbugs workshop in Swansea Central Library (Swansea, United Kingdom) on 25 August 2023. This was a free entry and free access event, advertised across the social media channels of both Superbugs and the Swansea Library network. The event consisted of a number of Superbugs stations already proven to engage and educate, such as a microscope station, display of bacterial agar plates, and ‘Grow your own microbes’ (Tyrrell et al., 2022) but also incorporating the new draft activities developed by the MSc students. Feedback was collected both through informal surveys at the individual stations and official questionnaires handed out to visitors (Table 1), based on feedback forms successfully used in previous projects (Tyrrell et al., 2022, 2024).

**Table 1:** Ratings of activities by visitors of the Superbugs event at Swansea Central Library.

Activity	How fun were the following activities?	How much did you learn from the following activities?
Grow your own microbes	4.3 ± 1.1 (*n* = 14)	4.5 ± 0.7 (*n* = 13)
Bacterial growth display	4.6 ± 0.7 (*n* = 14)	4.8 ± 0.4 (*n* = 13)
Microscopes	4.8 ± 0.6 (*n* = 13)	4.2 ± 1.0 (*n* = 14)
Microbial treasure hunt	4.6 ± 0.9 (*n* = 14)	4.8 ± 0.4 (*n* = 12)
Microbial card game	4.3 ± 0.5 (*n* = 9)	4.1 ± 0.7 (*n* = 10)
Superbugs website	3.8 ± 1.3 (*n* = 11)	4.3 ± 1.0 (*n* = 12)

Data shown are average scores ± standard deviations (number of submitted answers). Rating: (1) = ‘*Not at all*’, (2) = ‘*Not much*’, (3) = ‘*Neutral*’, (4) = ‘*A little*’, (5) = ‘*Very much*’.

### 2.4 Delivery of final form

Feedback collected from the Swansea Central Library event was used to make informed decisions on the content, design and overall efficacy of the designed materials to achieve the stated aims, and to mature them to their final form. On 22 September 2023, a final form workshop was delivered to Year 2 pupils (*n* = 42, split across two separate workshops) at Cefn Glas Infants School. This workshop consisted of a short introductory presentation, followed by delivery of five activities. Each class was split up into smaller groups, which took turns sequentially circling around each work station. Similar to the previous library event, this workshop consisted of three existing Superbugs stations (‘Microscope World’, ‘Grow Your Own Microbes’, and a handwashing demonstration; see Tyrrell et al., 2022), and the two new in-person activities developed as part of this current project. The MSc students returned to Cefn Glas for a second follow-up workshop on 24 September 2023. This involved providing pupils with an opportunity to observe what had grown from the individual agar plates (as part of the ‘Grow Your Own Microbes’ activity) and to take part in a quiz about what they had learnt in order to evaluate impact of the workshop intervention.

### 2.5 Impact evaluation

Following the workshop, further evaluatory data were collected through the provision of feedback questionnaires to the teachers involved (Tables 2, 3). In addition to the teachers, the MSc project students themselves were also asked to provide reflective feedback, focusing on what they had gained or learnt in terms of public engagement, and on their professional development as research scientists.

**Table 2:** Ratings of activities by participating teachers at Cefn Glas Infants School, Bridgend.

Activity	How fun were the following activities?	How informative were the following activities?
Grow your own microbes	5	5
Handwashing challenge	5	5
Microscopes	5	5
Microbial treasure hunt	4.75 ± 0.5	4.75 ± 0.5
Microbial card game	4.5 ± 0.58	4.25 ± 0.5
Follow-up session and quiz	4.75 ± 0.5	5

Numbers shown are average scores ± standard deviations from *n* = 4 submitted answers. Rating: (1) = ‘*Not at all*’, (2) = ‘*Not much*’, (3) = ‘*Neutral*’, (4) = ‘*A little*’, (5) = ‘*Very much*’.

**Table 3:** Feedback from individual teachers at Cefn Glas Infants School, Bridgend.

**Please provide any further comments on the overall performance and engagement from our project students**
*‘All students were welcoming and warm when speaking to the children. They tried to ensure that they pitched the lesson to the children’s ability. They used positive praise and feedback and the children were engrossed in all activities. Diolch yn fawr’.* ^*^
*‘All students were very approachable and explained things in an appropriate manner’.*
*‘The students were well prepared and well-resourced for the session. They tried their best to pitch the vocabulary at the learners’ level, not an easy task. In an ideal world it would have been more beneficial to the children to have longer than 20 min at a station. The children loved the feedback session, especially seeing the germs they had grown. Thank you for a fantastic introduction to your expertise!’*
**Do you have any further comments on elements of the workshop that you wish to add?**
*‘I think the children particularly enjoyed seeing how many germs had grown in the follow up session. The handwashing station also provided a very visual way to demonstrate the importance of hygiene’.*
*‘The children were very enthusiastic about all the activities. During a plenary at the end of the day it became apparent that the least favourite activity was the card game. When questioned they said they liked it but needed longer to play’.*
**What improvements would you suggest for future deliveries of this Superbugs workshop?**
*‘We wish you could have stayed longer! I think that the children would have benefitted from a longer session with you, but I understand the time restrictions of the workshop. We would welcome you back to work with the children anytime. Thank you for an engaging workshop. It was well organised and thought out. Diolch yn fawr’.* ^*^
*‘I found the workshop satisfactory in its current format’.*
*‘Longer activity sessions. The children loved the activities, they captured the children’s imaginations perfectly’.*

^*^‘Diolch yn fawr*’* means ‘Thank you very much*’* in Welsh.

## 3 Results

### 3.1 Information gathering and design of educational activities

An initial questionnaire focusing on in-person activities was sent out via Twitter/X to UK-based science teachers and gathered 14 responses from educators of varying level and background. This exercise provided the MSc BMS students with insights into the mechanics of current education on topics such as microbiology and personal hygiene (Supplementary Table S1). Whilst 71% of teachers indicated that they regularly integrated scientific topics into their everyday teaching, and 63% indicated they were at least *‘Somewhat confident’* in teaching health topics relating to microbiology and infection, there was still a strong appetite for resources/opportunities to help incorporate the teaching of health-related topics of infection and microbiology. When asked to rank modes of teaching delivery in terms of their effectiveness in improving understanding of new topics, *‘Hands-on experiments’* (50%) and *‘Competitions/games’* (43%) were ranked the most effective.

A second questionnaire was developed specifically for the creation of digital educational material (Supplementary Table S2). Here, across 10 science teachers recruited via Twitter/X, 90% believed that interactive online/digital tools are an effective way of engaging pupils, and that in addition to this, interactive games/puzzles are the most effective mode of engagement. Importantly for this project, 80% suggested their pupils had only little understanding of microbiology and infection science.

### 3.2 Development of educational activities

The questionnaires provided a strong directional guide for the development of new activities. Based on the information collected, two new challenge/game-based activities were designed.

#### 3.2.1 Activity 1: ‘Microbial Treasure Hunt’

**Figure 1:**
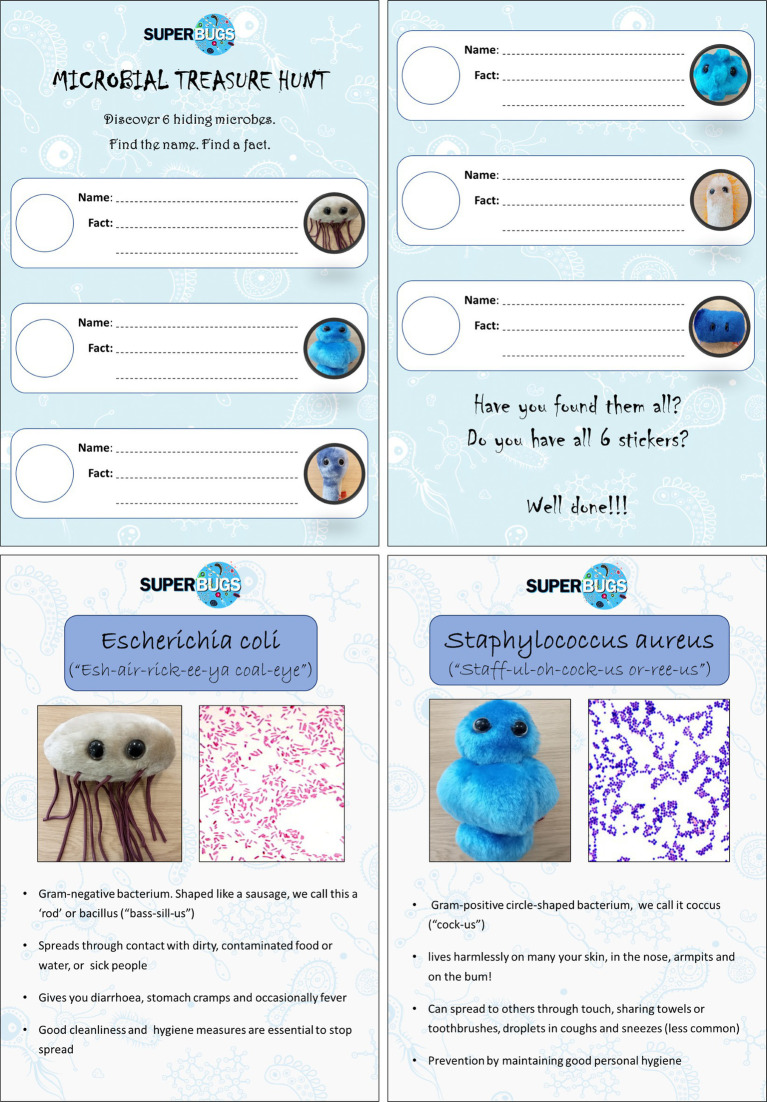
Materials produced for the Microbial Treasure Hunt. Top row, front and back pages of the treasure hunt sheet provided to each pupil. Bottom row, two examples of fact sheets being ‘hunted’.

Pupils were provided with a ‘Hunt Sheet’ (Figure 1) on which they had to collect names and facts of six common micro-organisms related to personal and food hygiene—*Escherichia coli*, *Staphylococcus aureus*, *Listeria monocytogenes, Clostridium difficile, Shigella dysenteriae*, and rhinovirus. The ‘Treasure’ was represented by a Giant Microbe cuddly toy^3^ and an accompanying information poster with key facts. Pupils had to find the ‘treasure’ and complete the treasure hunt sheet by writing down a selected fact, in the allotted time, to complete the challenge and win a small prize. Two examples of the fact sheets used can be seen in Figure 1.

#### 3.2.2 Activity 2: ‘Microbial Superhero/Supervillain’ card game

**Figure 2:**
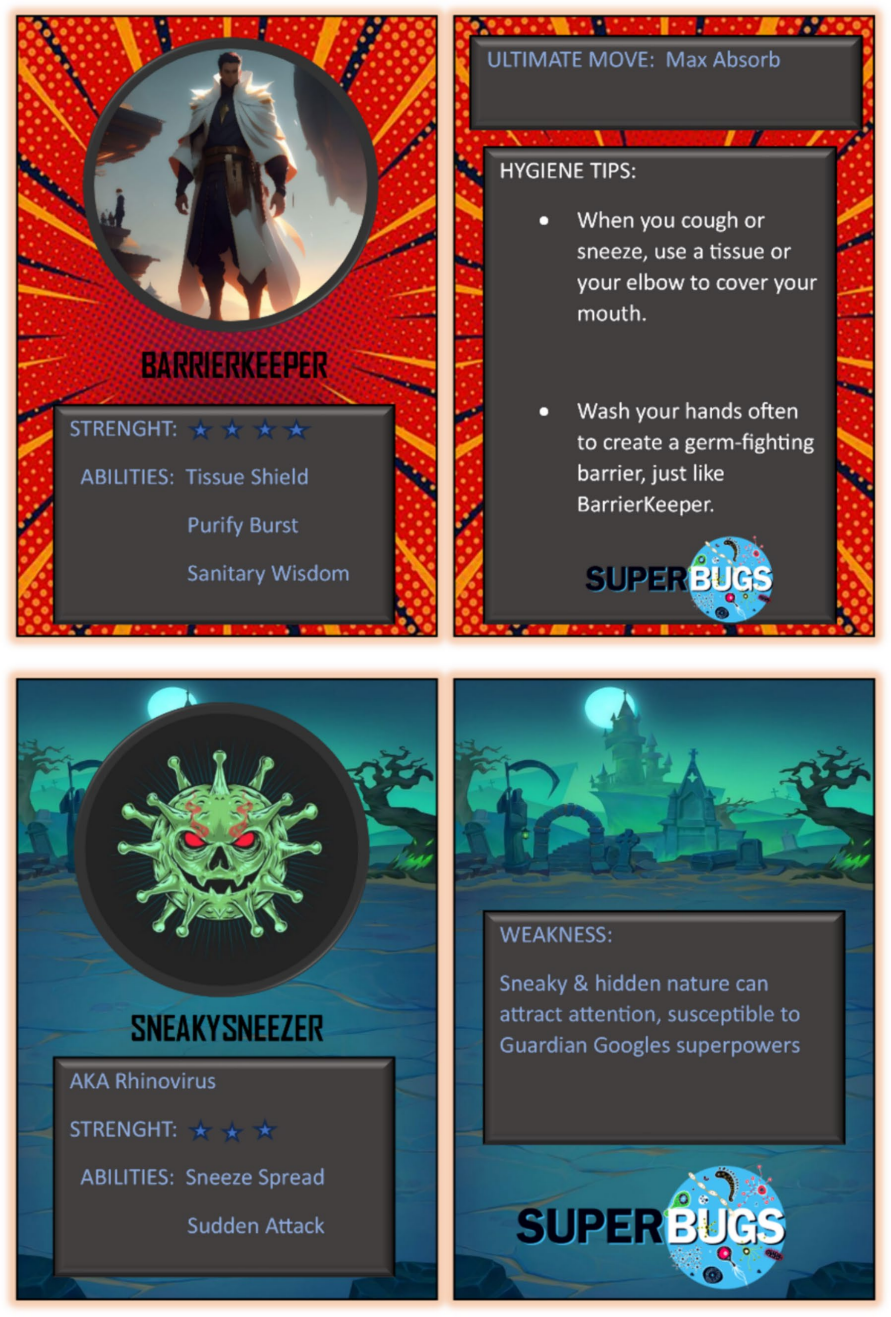
Materials produced for the Microbial Superhero/Villain card game. Examples show the front and back sides of cards from game representing ‘superheroes’ (top; strategies to prevent, contain or treat infections) and ‘supervillains’ (bottom; pathogens causing disease).

Pupils played as either Microbial ‘Superheroes’ or ‘Supervillains’. The game could be played either individually, one-on-one, or in teams of two. Each team of players was given six characters with specific abilities either complementary or antagonistic to members of the opposite team. For the six ‘Supervillains’, these powers would be focused on particular ways in which a pathogenic microbe would spread and/or cause infection. For the six ‘Superheroes’, powers were based around personal hygiene interventions. The first player would select a character and place down on the table. The aim was for the opposite players to select the correct member of your team to ‘wipe out’/kill the opposing team’s characters one at a time. Examples of the card game are shown in Figure 2.

**Figure 3:**
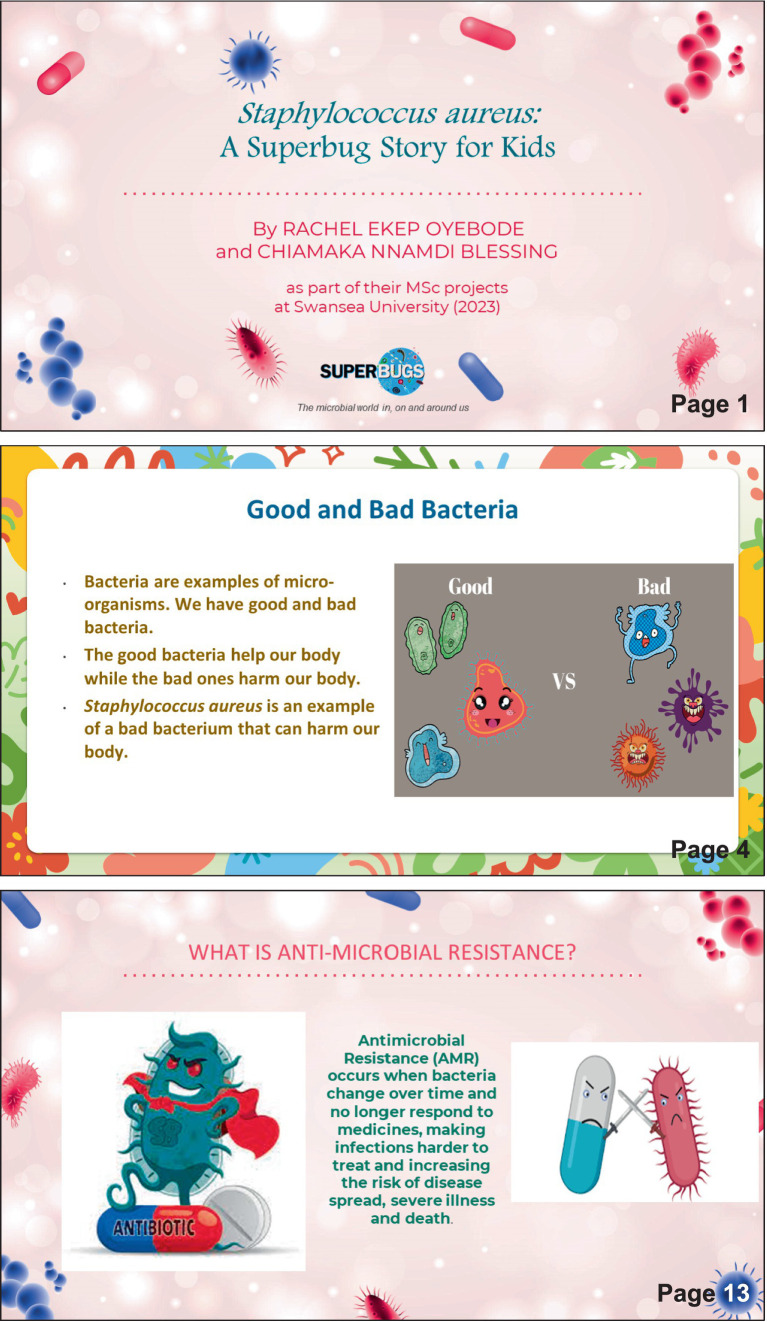
Materials produced for the booklet ‘*Staphylococcus aureus*: A Superbugs Story for Kids’. The full version can be downloaded from the Superbugs website (see text footnote 2).

Whilst questionnaire feedback indicated the utility of interactive games/puzzles for educational purposes, after a number of development steps it was decided that producing such material, of a high enough quality within the time constraints of a 12-week student project, was unrealistic. As such a decision was taken to change direction and focus on another element that was reflected in the feedback of both questionnaires: Real Life Stories. In doing so, an online booklet was created—a case study on a particular pathogen, *S. aureus* (Figure 3). Using images and infographics, it provided messaging along the same themes of personal and food hygiene.

### 3.3 Feedback from community-based workshop

Across the two-hour session in the Swansea Central Library, 67 visitors engaged with the Superbugs workshop. From these, 14 completed questionnaires were collected. Although this represents only 22% of visitors, many questionnaires were filled in as family units (children with parents/guardians), so the feedback reflected in these questionnaires represents a higher catchment than perhaps first thought. Visitors were asked to rank the different activities both in terms of how fun/engaging they were, and how informative. The treasure hunt was ranked highly in both categories, the card game somewhat lower (Table 1). The Superbugs website and the digital *S. aureus* story book, presented online on computers within the library, were ranked relatively low in the ‘fun’ category but were still considered quite informative. Overall, 80% of visitors rated their Superbugs experience as ‘very good’/‘excellent’. The event was a success in improving the knowledge of the participants on personal hygiene (87%), food hygiene (87%), micro-organisms (100%) and infections (73%).

### 3.4 Feedback from school workshop

Responses to the workshop delivered at Cefn Glas Infants School were overwhelmingly positive and enthusiastic. All activities delivered were ranked highly by the supervising teachers (Table 2); qualitative comments were full of praise for both the MSc students as facilitators and the workshop content (Table 3). In-class voting by the pupils showed that two of the new activities developed as part of this project, the Microbial Card Game (65%) and the Microbial Treasure Hunt (58%), were the most favoured amongst the pupils, with the other activities receiving 33–45% of the votes. Over 80% of pupils completed the microbial treasure hunt. This equates to them encountering, engaging and becoming familiar with the six chosen pathogens (Figure 1), and the information provided about them. This was reflected in the evaluation data collected. In the follow up quiz on the second visit, 30% of pupils could identify infections—in some cases the actual causative species—based on key facts such as characteristics of the infection, shape of the micro-organism or associated symptoms, and nearly 70% successfully identified potential reservoirs for infection transmission. This was a substantial increase from 0 and 45%, respectively, at the beginning of the first session 2 days prior.

### 3.5 Impact on MSc student reflections

Unanimously, all four MSc students indicated that as a result of their participation in Superbugs projects, they had more awareness of the nature of public engagement and its importance within the context of research and research communication, and as such would be more likely take part in public engagement projects in the future. On the perspective of research skills, all four MSc students now felt more confident in their ability to carry out independent literature research, in the quality of their scientific writing and in their ability to engage and communicate science (concepts, data and conclusions) to a variety of audiences, both peer and lay.

## 4 Discussion

We here give a succinct account of the latest project from the ‘Superbugs’ initiative — the development of educational material and activities aimed at improving the knowledge of young children on the topics of microbiology, infection biology and AMR. Previous iterations of Superbugs activities focused on delivering these aims in public spaces (Tyrrell et al., 2022) and replicating this experience through an educational website (Tyrrell et al., 2024). The current study aimed to design and evaluate educational materials for use as part of in-person workshops to be delivered directly to young pupils in schools, and the efficacy of delivering these through MSc students. We present pilot evidence that postgraduate research projects, underpinned by active and service learning, represent a valid and effective way of delivering impactful public engagement. In turn, the experience holds benefit for the MSc students not only in terms of their academic study and core scientific skills, but also their wider appreciation and confidence in being confident and effective engagers and science communicators.

### 4.1 Impact and quality of educational material produced

After thorough appraisal of the relevant literature, the specific topic of personal and food hygiene was selected as the focus for the research projects. Whilst not a topic that Superbugs has directly addressed in a significant manner before, there is certainly currency in this choice. Personal hygiene is a key determinant of public health and wellbeing of a society and the focus of much school-targeting public engagement. In 2021, UK Research and Innovation (UKRI) and the Food Standard Agency (FSA) jointly launched the ‘Citizen Science for Food Standards Challenge’ aimed at using publicly involved strategies to investigate challenges in food standards and hygiene. A report describing the findings of this programme was published recently (Oakden et al., 2023). The need for such educational interventions was highlighted in the data collected by the student-designed questionnaires. In this current study, 71% of educators indicated that they taught and integrated topics of microbiology, infection and hygiene into their teaching, but only 50% indicated that there was a good understanding of these topics amongst their pupils. Thus, there is a clear disparity between the amount of teaching, and the impact of teaching, on these topics. It should be noted that information on the geographical location of the teachers completing the student’ initial, fact-finding questionnaires was not collected. This is pertinent and potentially limits the wider interpretation of the results as curriculum content varies between Wales and other parts of the United Kingdom.

The treasure hunt and card game activities were well received both in terms of how fun and engaging they were, and how they educated and informed. There was little difference between the rating when comparing the feedback from the different events at which the activities were presented (Swansea Central Library and Cefn Glas Infants School). To our knowledge this is the first published evaluation of a treasure/scavenger hunt-based game educating on microbiology and micro-organisms. There is precedent for the use of card games being successful in obtaining positive public education, to varying degrees of complexity. Callahan et al. (2019) successfully used card games to educate and advance conservation and biodiversity. Other examples can be seen in the topics of microbiology (Tarín-Pelló et al., 2022), genetics (Vun et al., 2013), and immunology (Su et al., 2017).^4^

Less favourable reviews were obtained for the online story booklet presented at the library event. However, upon reflection and anecdotal feedback this was most likely due to the distraction of many hands-on activities also being available as opposed to the quality or attractiveness of the material on offer. How to successfully deliver a hybrid event of hands-on and online/digital offerings will be investigated in future deliveries of Superbugs community events. There is certainly potential that ‘*S. aureus*, a Superbug Story for Kids’ may represent an opportunity for legacy engagement — material provided to teachers following a Superbugs workshop, for them to continue the themes of the workshop, but integrated within their own teaching. The impact and viability of this theory will be investigated in future Superbugs projects.

### 4.2 Reflection on pedagogy and impact upon students

From a pedagogical standpoint, service learning in the context of this project may be defined as ‘applied active learning’. The pedagogical approach of service learning has been shown to be academically beneficial to partaking students, both within the field of microbiology (Webb, 2016; Fatton et al., 2021) and beyond in wider science, technology, engineering and mathematics (STEM) subjects (Freeman et al., 2014). The MSc students who undertook this project did exhibit this benefit, reporting an increase in confidence of key scientific skills such as reviewing of the literature, scientific writing and presenting, that are a cornerstone of developing competent independent scientists. In line with Mtawa et al. (2019), service learning as applied here may have more far-reaching benefits beyond just skills development, possibly even improving employability potential—arguably even more so than more traditional laboratory-based research projects.

These benefits were engendered by an independent and peer-led approach to the supervision of the students. They were responsible for the topic of choice, originating the ideas on activities and material, designing and the ultimate delivery of said activities. They were encouraged to discuss and critically analyse different ideas and approaches to achieving the project aims. The benefits of peer-led learning are well documented (Byl et al., 2016; Stigmar, 2016), were evidently effective in skills development in this project, and correlate neatly with service learning. However, the experience was not without areas highlighted for future improvement. As described, the first stage of the research project was self-driven, independent study by the students. The students were expected to familiarise themselves with the concepts and underlying philosophies of public engagement, and the literature of this in relevant areas. Student feedback suggested that this was very challenging, one where they felt more direct supervision and support was necessary. This was not due to lack of skills in finding and reading published literature, but a lack of accessibility to the subject. The students lacked confidence and conviction in knowing where to begin and what to read in such a broad topic as alien and novel to them as public engagement research. This we believe is an indictment of how little science communication, public engagement and public involvement are addressed in undergraduate and postgraduate STEM education (Stevens, 2011), and certainly one area we will focus support on more in future iterations of student-driven Superbugs projects.

### 4.3 Advantages to public engagement

The intellectual impact of student-driven projects described within, for both the target stakeholders and the students themselves has been well documented above. It comes as no surprise that, whilst increasing in reputation, public engagement is still an undervalued sector within academia and research (Varner, 2014; Eberl et al., 2023). This is in part due to a lack of academic currency (Davies, 2013) but may also be due to a simple disconnect to how scientists perceive the importance of their work in the minds of the public (Llorente et al., 2019). There is no doubt now however, that the strategic role and impact of public engagement is expanding (Mahony and Stephansen, 2017; Copley, 2018). As such, more volunteers will be required to populate ever-increasing opportunities. Crudely, student-led research projects built around public engagement may be seen as a way of making use of an ‘underutilised’ workforce. However, as we have shown here, the benefits may be more widely altruistic. All participating students stated they would be more inclined to become involved in public engagement in the future and would have more confidence in doing so. This suggests a way of breeding the next generation of public engagement professionals and enthusiasts (Mills and Chandrangsu, 2023; Robredo et al., 2023). The very nature of public engagement being delivered through the medium of a research project provides academic output, as evidenced here, which adds further incentivisation for all involved beyond the initial remit of the project.

### 4.4 Looking ahead

This current work enabled evaluation of the viability and efficacy of delivering public engagement projects through postgraduate student research projects. In agreement with the work of others, we have proven that this is possible and that service learning can indeed produce high quality, impactful public engagement (Webb, 2016). The next steps will now aim to more stringently evaluate the activities and workshop delivery in schools across Wales, and beyond. Is the same level of positive messaging and impact translatable across different age ranges, across schools in areas of varying socio-economic standing? The pursuits will allow us to further understand the mechanics of truly powerful AMR messaging and public engagement, a core value at the heart of everything Superbugs aspires to achieve.

## Data availability statement

The original contributions presented in the study are included in the article/Supplementary material, further inquiries can be directed to the corresponding authors.

## Ethics statement

This study did not classify as research involving human subjects, human material or human data, and as such did not require approval by an appropriate ethics committee. The individuals involved in this project were public involvement and engagement participants, and not individuals recruited into a research study. All individuals provided verbal consent to take part in this study.

## Author contributions

JMT: Conceptualisation, Data curation, Formal analysis, Funding acquisition, Methodology, Project administration, Resources, Supervision, Validation, Visualisation, Writing – original draft, Writing – review and editing. HUA: Data Curation, Formal analysis, Investigation, Methodology, Writing – original draft, Writing – review and editing. VNG: Data curation, Formal analysis, Investigation, Methodology, Writing – original draft, Writing – review and editing. RO: Data curation, Formal analysis, Investigation, Methodology, Writing – original draft, Writing – review and editing. CNB: Data curation, Formal analysis, Investigation, Methodology, Writing – original draft, Writing – review and editing. SH: Validation, Visualisation, Writing – original draft, Writing – review and editing. ME: Conceptualisation, Funding acquisition, Methodology, Project administration, Resources, Co-supervision, Validation, Visualisation, Writing – original draft, Writing – review and editing.

## Footnotes

^1^ ukhsa.blog.gov.uk/2023/11/15/the-fight-against-antimicrobial-resistance-progress-and-challenges

^2^ www.superbugs.online

^3^ www.giantmicrobes.com

^4^ www.open.ed.ac.uk/supercytes
