# Supplementary material for: Combining postgraduate research training, public engagement, and primary school science education—a Superbugs Master (MSc) class
Source: Front Microbiol. 2024 May 31;15:1380045. doi: 10.3389/fmicb.2024.1380045 (PMC11176509; doi:10.3389/fmicb.2024.1380045)
Supplement: Supplementary file 1 [file Table_1.DOCX]

Combining postgraduate research training, public engagement and primary school science education
 – A Superbugs Master (MSc) class

Jon M Tyrrell^1^, Haritha Udayan Ayanikkad^1^, Vasudev Nalleppillil-Gopakumar^1^, Rachel Oyebode^1^, Chiamaka Nnamdi Blessing^1^, Sarah Hatch^2^, Matthias Eberl^3,4^

*^1^Institute of Life Science, School of Medicine, Swansea University, Swansea, UK; ^2^Public Involvement and Engagement Team, School of Medicine, Cardiff University, Cardiff, UK; ^3^Division of Infection and Immunity, School of Medicine, Cardiff University, Cardiff, UK; ^4^Systems Immunity Research Institute, Cardiff University, Cardiff, UK*

*** Correspondence:**

Jon Tyrrell: [j.m.tyrrell@swansea.ac.uk](mailto:j.m.tyrrell@swansea.ac.uk) ; Matthias Eberl: [eberlm@cf.ac.uk](mailto:eberlm@cf.ac.uk)

**Supplementary Information**

**Supplementary Table S1. Questionnaire 1 — In-person Activities Project.** Responses from 14 UK-based science teachers.

| **Question 1: Which age group do you teach?** | |
| --- | --- |
| *Options* | *Responses* |
| 1 - 5 years old | 14.3% (2/14) |
| 6 – 9 years old | 42.9% (6/14) |
| 10 – 16 years old | 71.4% (10/14) |
| **Question 2: If teaching, what subject(s) do you currently teach?** | |
| *Options* | *Responses* |
| Science | 78.6% (11/14) |
| Mathematics | 14.3% (2/14) |
| English/languages | 7.1% (1/14) |
| Social Studies/History | 7.1% (1/14) |
| Art/Creative Expression | 7.1% (1/14) |
| Physical Education | 7.1% (1/14) |
| Other | 14.3% (2/14) |
| **Question 3: How often do you integrate scientific topics into your daily teaching/engagement with young people?** | |
| *Options* | *Responses* |
| Regularly, in multiple subjects | 71.4% (10/14) |
| Occasionally, in specific subjects | 21.4% (3/14) |
| Rarely, in limited subjects | 7.1% (1/14) |
| Never, I focus on my subject specifically | 0% |
| **Question 4: In your experience, how can scientific topics be included alongside non-science subjects effectively?** | |
| *Options* | *Responses* |
| Through interdisciplinary projects | 35.7% (5/14) |
| Using real life examples/stories | 92.9% (13/14) |
| Collaborating with science educators | 57.1% (8/14) |
| Other | 0% |
| **Question 5: How Comfortable do you feel discussing scientific topics, such as hygiene and health risks, when they arise in non science subjects?** | |
| *Options* | *Responses* |
| Very comfortable | 78.6% (11/14) |
| Somewhat comfortable | 21.4% (3/14) |
| Neutral | 0% |
| Somewhat uncomfortable | 0% |
| Very uncomfortable | 0% |
| **Question 6: Do you think adding scientific topics of public importance to non-scientific topics can enhance student’s overall understanding and engagement of the topic?** | |
| *Options* | *Responses* |
| Yes | 100% (14/14) |
| No | 0% |
| Not sure | 0% |
|  |  |

| **Question 7: Would you be interested in resources and/or professional development opportunities that help incorporate science into non-science topics?** | |
| --- | --- |
| *Options* | *Responses* |
| Yes | 64.3% (9/14) |
| No | 7.1% (1/14) |
| Unsure | 28.6% (4/14) |
| **Question 8: How confident do you feel teaching your students about health topics such as infections etc?** | |
| *Options* | *Responses* |
| Very confident | 42.9% (6/14) |
| Somewhat confident | 28.6% (4/14) |
| Neutral | 21.4% (3/14) |
| Somewhat unconfident | 7.1% (1/14) |
| Very unconfident | 0% |
| **Question 9: Have you previously discussed/taught topics relating to microbiology, personal/public hygiene and related health risks?** | |
| **Options** | **Responses** |
| Yes, regularly | 42.9% (6/14) |
| Yes, occasionally | 35.7% (5/14) |
| Yes, but rarely | 14.3% (2/14) |
| Never | 7.1% (1/14) |
| **Question 10: Please provide further information if you answered ‘Yes’, ‘Occasionally’ or ‘Rarely’ to Question 9.** | |
| *‘Mainly in the relevant topic of the curriculum health and diseases. Rush assessments/hygiene for practicals’* | |
| *‘Microbiology post-doc who did school outreach’* | |
| *‘Our topic is Health & Wellbeing for September. We cover most of these on a daily basis.’* | |
| *‘I teach science by mainly chemistry. This topic doesn’t come up often’* | |
| *‘Answering as a parent also, I work in clinical microbiology so my children are regularly reminded of the consequences of poor hygiene, probably with way too much gory detail’* | |
| *With the Michigan model regarding Health & reproductive health. I also bring microbiology and health up quite a bit in my regular teaching, especially while I am coaching wr’estling’* | |
| *‘I teach biology at KS3 level to SEN students’* | |
| **Question 11: How well do you think your students understand microbiology, and their role in causing health risks?** | |
| *Options* | *Responses* |
| Extremely well | 0% |
| Somewhat well | 50.0% (7/14) |
| Neutral | 35.7% (5/14) |
| Somewhat not well | 7.1% (1/14) |
| Extremely not well | 7.1% (1/14) |
| **Question 12: On a scale of 1 (not at all) to 5 (very well), how well do you think your pupils understand the COVID-19 pandemic**  *Options Responses* | |
| 1 | 0% |
| 2 | 0% |
| 3 | 78.6% (11/14) |
| 4 | 14.3% (2/14) |
| 5 | 7.1% (1/14) |

| **Question 13: How do you think your pupils would react to learning about health and hygiene, involving microbiology & micro-organisms** | |
| --- | --- |
| *Options* | *Response* |
| Extremely interested | 50.0% (7/14) |
| Somewhat interested | 50.0% (7/14) |
| Neutral | 0% |
| Somewhat not interested | 0% |
| Extremely not interested | 0% |
| **Question 14: How do you typically deliver scientific lessons/information to your students/young people?** | |
| *Options* | *Responses* |
| Traditional lessons/presentations | 35.7% (5/14) |
| Interactive group activities/discussions | 92.9% (13/14) |
| Hands-on experiments & demonstrations | 78.6% (11/14) |
| Multimedia resources (videos etc) | 50.0% (7/14) |
| **Question 15: Please rank the below options in order of the effectiveness in engaging your pupils and improving their understanding of new concepts and topics** | |
| *Ranking* | *Options and Rate of Ranking* |
| Most effective | Hands on experiments (50.0%)  Competitions/games stories (42.9%)  Real life stories (21.4%) |
| Least Effective | Traditional lessons/presentations (5.17%)  Competitions/games (28.6%)  Multimedia resources (7.1%)  Real life stories (7.1%) |
| **Question 16: How important do you think it is to make scientific topics, such as personal/food hygiene and related health risks, relatable and accessible to your pupils** | |
| *Options* | *Responses* |
| Extremely important | 100% (14/14) |
| Somewhat important | 0% |
| Neutral | 0% |
| Somewhat not important | 0% |
| Extremely not important | 0% |
| **Question 17: Would you be interested in professional development opportunities/workshops focused on teaching microbiology and related topics to pupils?** | |
| *Options* | *Responses* |
| Yes | 50.0% (7/14) |
| No | 14.3% (2/14) |
| Maybe, if it aligns to my teaching goals | 35.7% (5/14) |

**Supplementary Table S2. Questionnaire 2 — in support of Digital/Online Material.** Responses from 10 UK-based science teachers.

| **Question 1: Which of the below options best described you?** | |
| --- | --- |
| *Options* | *Responses* |
| Teacher | 90% (9/10) |
| Parent | 0% |
| Both | 10% (1/10) |
| Neither | 0% |
| **Question 2: To what degree has your child/pupil been educated on microbiology & infection** | |
| *Options* | *Responses* |
| Regularly/large amount | 10% (1/10) |
| Rarely/little | 90% (9/10) |
| Don’t Know | 0% |
| **Question 3: To what level do you think your child/pupil has an understanding of microbiology and infection?** | |
| *Options* | *Responses* |
| A high level of understanding | 10% (1/10) |
| A little understanding | 80% (8/10) |
| No understanding | 10% (1/10) |
| Don’t know | 0% |
| **Question 4: Following recovery from the COVID-19 Pandemic, how is your teaching now delivered?** | |
| *Options* | *Responses* |
| All in-person activities | 60% (6/10) |
| Hybrid (mix of in-person and digital) | 40% (4/10) |
| Primarily online/digital tools | 0% |
| **Question 5: Do you think an interactive online/digital education tools are an effective way of engaging students in new scientific topics/concepts?** | |
| *Options* | *Responses* |
| Yes | 90% (9/10) |
| No | 0% |
| Don’t know | 10% (1/10) |
| **Question 6: When using online/digital education platforms, what method do you find the most effective in engaging pupils and helping them understand new concepts?** | |
| *Options* | *Responses* |
| Text & pictures | 30% (3/10) |
| Interactive games/quizzes | 100% (10/10) |
| Artwork/creative tasks | 50% (5/10) |
| Real life stories | 80% (8/10) |
| Multimedia visuals/videos | 60% (6/10) |
| Other | 0% |
